# The P2X7 Receptor Mediates *Toxoplasma gondii* Control in Macrophages through Canonical NLRP3 Inflammasome Activation and Reactive Oxygen Species Production

**DOI:** 10.3389/fimmu.2017.01257

**Published:** 2017-10-12

**Authors:** Aline Cristina Abreu Moreira-Souza, Cássio Luiz Coutinho Almeida-da-Silva, Thuany Prado Rangel, Gabrielle da Costa Rocha, Maria Bellio, Dario Simões Zamboni, Rossiane Claudia Vommaro, Robson Coutinho-Silva

**Affiliations:** ^1^Immunobiology Program, Biophysics Institute Carlos Chagas Filho, Federal University of Rio de Janeiro, Rio de Janeiro, Brazil; ^2^Parasitology and Cell Biology Program, Biophysics Institute Carlos Chagas Filho, Federal University of Rio de Janeiro, Rio de Janeiro, Brazil; ^3^Department of Immunology, Institute of Microbiology Paulo de Goes, Federal University of Rio de Janeiro, Rio de Janeiro, Brazil; ^4^Department of Cell Biology, Ribeirão Preto Medical School, University of São Paulo, São Paulo, Brazil

**Keywords:** P2X7 receptor, *Toxoplasma gondii*, NLRP3 inflammasome, caspase-1, caspase-11, IL-1β, reactive oxygen species

## Abstract

*Toxoplasma gondii (T. gondii)* is the protozoan parasite that causes toxoplasmosis, a potentially fatal disease to immunocompromised patients, and which affects approximately 30% of the world’s population. Previously, we showed that purinergic signaling *via* the P2X7 receptor contributes to *T. gondii* elimination in macrophages, through reactive oxygen species (ROS) production and lysosome fusion with the parasitophorous vacuole. Moreover, we demonstrated that P2X7 receptor activation promotes the production of anti-parasitic pro-inflammatory cytokines during early *T. gondii* infection *in vivo*. However, the cascade of signaling events that leads to parasite elimination *via* P2X7 receptor activation remained to be elucidated. Here, we investigated the cellular pathways involved in *T. gondii* elimination triggered by P2X7 receptor signaling, during early infection in macrophages. We focused on the potential role of the inflammasome, a protein complex that can be co-activated by the P2X7 receptor, and which is involved in the host immune defense against *T. gondii* infection. Using peritoneal and bone marrow-derived macrophages from knockout mice deficient for inflammasome components (NLRP3^−/−^, Caspase-1/11^−/−^, Caspase-11^−/−^), we show that the control of *T. gondii* infection *via* P2X7 receptor activation by extracellular ATP (eATP) depends on the canonical inflammasome effector caspase-1, but not on caspase-11 (a non-canonical inflammasome effector). Parasite elimination *via* P2X7 receptor and inflammasome activation was also dependent on ROS generation and pannexin-1 channel. Treatment with eATP increased IL-1β secretion from infected macrophages, and this effect was dependent on the canonical NLRP3 inflammasome. Finally, treatment with recombinant IL-1β promoted parasite elimination *via* mitochondrial ROS generation (as assessed using Mito-TEMPO). Together, our results support a model where P2X7 receptor activation by eATP inhibits *T. gondii* growth in macrophages by triggering NADPH-oxidase-dependent ROS production, and also by activating a canonical NLRP3 inflammasome, which increases IL-1β production (*via* caspase-1 activity), leading to mitochondrial ROS generation.

## Introduction

*Toxoplasma gondii* is a parasitic protozoan from the phylum Apicomplexa and the etiologic agent of toxoplasmosis, an important disease that affects 30% of the world’s population (1, 2). Toxoplasmosis can be transmitted by organ transplantation, blood transfusion or congenital infection, due to the parasite’s ability to cross biological barriers (3, 4). In most cases, *T. gondii* causes asymptomatic disease, but toxoplasmosis can be fatal in immunocompromised patients, such as those with HIV/AIDS, cancer, and organ transplants (2). Also, severe ocular disease and multiple organ failure in immunocompetent individuals have been observed in endemic countries, due to the circulation of atypical parasite strains (5, 6). The current therapy for toxoplasmosis is based on drug combinations such as sulfadiazine/pyrimethamine, which reduce parasite replication, controlling host damage and symptoms; however, combination therapy causes important side effects and does not eliminate the resistant *T. gondii* cysts (7, 8).

*T. gondii* is an obligate intracellular parasite, able to infect virtually all nucleated cell types in a wide variety of hosts (2). It has been proposed that *T. gondii* utilizes innate immune cells, like macrophages and dendritic cells, to migrate to preferential infection sites, such as the immune “shielded” environment of the central nervous system, establishing lifelong chronic infection (9, 10). The parasite survives inside host cells by overcoming many host antimicrobial mechanisms, including reactive oxygen species (ROS) and nitric oxide (NO) production, lysosome fusion (to the parasitophorous vacuole), host cell death induction, and the secretion of pro-inflammatory cytokines and chemokines (11). Concomitantly, *T. gondii* triggers the activation of host cell anti-inflammatory transcription factors, inducing the over expression of receptors involved in host cell migration to the brain, which characterizes the chronic phase of toxoplasmosis (12). The ability to subvert immune system mechanisms and the fast establishment of the latent (chronic) phase of the disease directly jeopardize treatment for toxoplasmosis.

Inflammasomes are multimeric protein complexes that produce active caspase-1 by cleavage of procaspase-1 (13), and whose activation triggers maturation of IL-1β/IL-18 and pyroptosis, a type of inflammatory cell death (14). The inflammasome NLRP3 are formed by nucleotide-binding domain and leucine-rich repeat-containing (NLR) proteins, an apoptosis-associated spec-like protein containing a caspase-recruitment domain (ASC) protein adaptor and an inactive zymogen, procaspase-1 (Casp-1) (14). In mice, activation of the NLRP3 inflammasome requires two signals. First, recognition of a pathogen-associated molecular pattern by cell surface molecules known as pattern recognition receptors (PRRs) leads to the transcription of pro-inflammatory cytokines (15). Then, activation of the cytosolic PRRs results in the oligomerization of inflammasome components and procaspase-1 cleavage into active caspase-1 (13). The active caspase-1 subsequently cleaves the pro-inflammatory IL-1-family cytokines into their bioactive forms IL-1β/IL-18 and induces pyroptosis (14). Inflammasomes involved in caspase-1 activation are known as canonical inflammasomes, while those related to caspase-11 (in mice) or caspase-4/5 (in humans) are known as non-canonical inflammasomes (16, 17). Activation of the canonical NLRP3 inflammasome is trigged by cytosolic stress conditions such as K^+^ efflux, leakage of lysosome components, mitochondrial damage, and ROS production (14, 18). Interestingly, the P2X7 nucleotide receptor activation involved in the elimination of different intracellular parasites, such as *Leishmania amazonensis* (19), *Porphyromonas gingivalis* (20), *Mycobacterium tuberculosis* (21–24), and *T. gondii* (25–27), induces most of these stress conditions and, thus, is capable of activating the NLRP3 inflammasome (28–30). Polymorphisms in the human P2X7 receptor are directly associated with host susceptibility to congenital or acquired toxoplasmosis, in immunocompetent patients (25, 31). Also, P2X7 absence or disruption of P2X7 receptor function increases toxoplasmosis severity in murine infection with virulent (RH, type I) or non-virulent (Me-49, type II) strains of *T. gondii* (27, 32).

Our group demonstrated that P2X7 receptor activation by extracellular ATP (eATP) in *T. gondii*-infected macrophages contributes to parasite elimination by ROS production and lysosome fusion with the parasitophorous vacuole (26). We also showed that the P2X7 receptor is an important activator of the anti-parasitic pro-inflammatory response that occurs in early *T. gondii* infection *in vivo* (32). However, the cascade of intracellular events trigged by purinergic signaling—which results in parasite control in early infection—remains to be elucidated.

In this work, we used macrophages from knockout mice lacking inflammasome components to examine the hypothesis that the inflammasome is involved in *T. gondii* infection control *via* eATP and the P2X7 receptor. We also studied the roles of ROS production and IL-1β secretion induced by P2X7 receptor in infected cell. Our combined data provide an overall model of the cellular pathways involved in *T. gondii* infection control mediated by the P2X7 receptor, in murine macrophages.

## Materials and Methods

### Reagents

Adenosine-5’-triphosphate (ATP), *N*-acetyl cysteine (NAC), penicillin, streptomycin, HEPES, Mito-TEMPO, apocynin, carbenoxolone, paraformaldehyde, and bovine serum albumin were purchased from Sigma Aldrich (USA). Fetal bovine serum (FBS) was from Gibco/Life Technologies (USA). Z-YVAD and recombinant IL-1β were from R&D Systems (USA).

### Mice

The following mouse strains were used in this work: Swiss CF1, C57BL/6 (wild-type strain), P2X7^−/−^ (originally from the Jackson Laboratory), Caspase-11^−/−^, Caspase1/11^−/−^ (Genentech, USA), and NLRP3^−/−^ mice generated on the C57BL/6 background (8–12 weeks old) were obtained from the Isogenic Breeding Unit at Ribeirão Preto Medical School, University of São Paulo, Ribeirão Preto, Brazil. Mice were maintained at the Animal House for Transgenic Mice of the Federal University of Rio de Janeiro (UFRJ), at 22°C in a 12-h light/dark cycle. Mice aged between 8 and 12 weeks were used in all experiments. The procedures for the care and use of animals were according to the guidelines of the Brazilian College of Animal Experimentation (COBEA). All efforts were made to minimize animal suffering and to reduce the number of animals used in this study. This study was approved and followed all the guidelines established by the Ethics Committee on the Use of Animals (CEUA) of the Biophysic Institute Carlos Chagas Filho (IBCCF, UFRJ, no. 082/15).

### Parasites

*T. gondii* tachyzoites from the RH strain were maintained in Swiss CF-1 mice as previously described in Ref. (33). Briefly, 5-week-old mice were infected intraperitoneally with 10^6^ tachyzoites. After 72 h of infection, parasites were harvested from the peritoneal cavity by PBS washing and then collected by centrifugation at 1,000 × *g* for 10 min. Harvested parasites were counted in a hemocytometer for experimental infections or for the next passage in mice.

### Bone Marrow-Derived Macrophages (BMDMs) and Peritoneal Macrophages

Bone marrow-derived macrophages were generated using L929 cell conditioned media (LCCM) as a source of macrophage colony-stimulating factor, as previously described (34, 35). RPMI containing 100 U/mL penicillin, 100 µg/mL streptomycin, and 1 mM sodium pyruvate was used throughout the procedure. In brief, fresh bone marrow from C57BL/6, NLRP3^−/−^, P2X7^−/−^, Caspase-11^−/−^, and Caspase-1/11^−/−^ mice were obtained and 5 × 10^6^ cells were plated in the petri dishes in RPMI with 20% FBS and 30% LCCM. On day 3 after plating, fresh medium was added to the cultures. On day 7 after plating, cells were harvested, washed with PBS, counted and plated in RPMI with 10% FBS and 5% LCCM, for cytokine detection assays (in 6-well plates, at 2 × 10^6^ cells/well) or light microscopy analysis (in 24-well plates with 13 mm coverslips, at 2 × 10^5^ cells/well). Cells were then incubated at 37°C for at least 18 h before the start of experiments. All experimental infections and treatments were performed in RPMI with 10% FBS (but without LCCM). Macrophage differentiation to >95% purity was confirmed by flow cytometry as described previously (35), before the start of each experiment.

Peritoneal macrophages were collected as previously described (33). Briefly, peritoneal exudates from C57BL/6 mice were obtained by washing the peritoneal cavity with 10 mL of sterile PBS. Peritoneal cells were counted by light microscopy, resuspended in DMEM medium, and 2 × 10^5^ cells were plated in 24-well plates with 13-mm coverslips for light microscopy analyzes. Cells were left to adhere for 1 h, at 37°C (and 5% CO_2_), and then non-adherent cells were removed by PBS washes before “supplemented DMEM” (DMEM with 10% FBS, 100 U/ml penicillin, 100 µg/ml streptomycin, and 10 mM HEPES) was added to cultures.

### Infection Assays

Cells were infected in supplemented DMEM medium, at 37°C (and 5% CO_2_). BMDMs or peritoneal macrophages were infected at a ratio of 3:1 tachyzoites to host cells for 2 h at 37°C (and 5% CO_2_), and non-internalized parasites were removed by PBS washes. Where required, cells were then incubated for 30 min in medium containing 3 mM eATP and/or 1 ng/mL IL-1β. In some experiments, cells were pretreated for 40 min with 10 mM NAC, 2 µM Z-YVAD, 1 µM Apocynin, 50 µM carbenoxolone, or 10 nM Mito-TEMPO, before ATP or IL-1β treatment. Then, cells were maintained at 37°C (and 5% CO_2_). After 18 h post-infection, aliquots of culture supernatants were collected and kept at −20°C, for IL-1β quantification (see “ELISA”) and cultures were processed for light microscopy, as described below.

### Light Microscopy

Infected cells were fixed in 4% paraformaldehyde and stained with Panoptic (“Panotico Rapido kit,” LaborClin, Brazil) following the manufacturer’s instructions. A minimum of 300 cells/sample were evaluated by light microscopy, in a Zeiss microscope (manufacturer). As previously described (33), the percentage of infection and infection index, which represents the overall infection load, were determined using the formula:

$$\%\text{ of infection}=\frac{\text{iC}\times 100}{\text{TotalC}}$$

$$\text{Infection Index}=\frac{\left\{\%\text{ of infection}\times \text{IntP}\right\}}{\text{TotalC}}÷100$$

where iC is number of infected cells; totalC is the total number of cells; and IntP is the number of intracellular parasites.

### Cytokine Assay—ELISA

IL-1β levels were quantified in supernatant samples from infected BMDM cultures (see Infection Assays), using the Mouse IL-1 beta/IL-1F2 DuoSet ELISA #DY401 kit, according to the manufacturer’s instructions (R&D Systems).

### ROS Assay

To estimate ROS production, cells were plated (at a density of 2 × 10^5^ cells/well) in opaque-black 96-well plates with a transparent and flat bottom. After 2 h of infection with *T. gondii*, cells were kept untreated or were subjected to one or both of the following treatments, in the presence of 2 µM of H_2_DCFDA: 10 mM NAC for 30 min, followed by 3 mM ATP for up to 60 min. Fluorescence was measured at 40, 50, and 60 min of exposure to ATP, using a Spectra Max^3^ spectrophotometer (Molecular Devices), at 37°C, at excitation and emission wavelengths of 490 and 520 nm, respectively.

### Statistical Analyses

A two-tailed *t*-test was used for comparisons of two groups, while multiple comparisons were performed by one-way ANOVA followed by Tukey’s post-test. All statistical analyses were performed using Graph Pad Prism 5 (La Jolla, CA, USA).

## Results

### P2X7 Receptor Activation Promotes *T. gondii* Control in Macrophages, *via* Inflammasome Activation, Caspase-1 and ROS Production

In a previous study, our group demonstrated that P2X7 receptor activation *via* eATP reduces *T. gondii* infection burden in peritoneal macrophages and J774.G8 cells (26). However, the mechanism of P2X7-induced *T. gondii* infection control in these cells remained unclear. On the other hand, eATP is capable of activating the inflammasome in infected cells (30) and promotes pathogen elimination in different models of infection (36). Therefore, we investigated whether *T. gondii* elimination in infected cells *via* eATP treatment was dependent on inflammasome activation.

**Figure 1:**
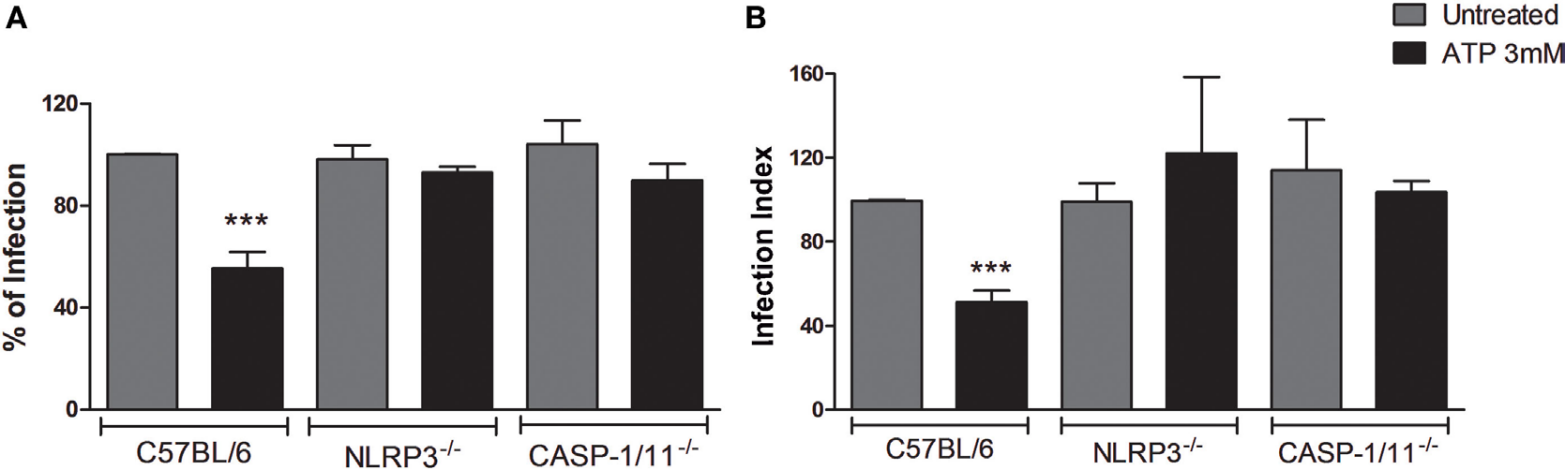
Extracellular ATP (eATP)-induced *T. gondii* control is dependent on NLRP3 and caspase-1/11 molecules. Bone marrow-derived macrophages from C57BL/6 (WT), NLRP3^−/−^ or Caspase-1/11^−/−^ mice were infected with *T. gondii* tachyzoites for 2 h and kept untreated, or were treated with 3 mM eATP for 30 min. Then, cells were incubated at 37°C for a total of 18 h, and stained with Panoptic, for light microscopy quantification of the percentage of infection **(A)** and the infection index **(B)**. Treatment with eATP reduced the percentage of infected cells and the infection index in wild-type (C57BL/6) macrophages, but not in the knockout cells. Normalized data represent mean ± SEM of three independent experiments performed in triplicates. ***p ≤ 0.001 vs. untreated (by two-tailed *t*-test).

To address this question, we infected BMDMs from wild-type C57BL/6 mice, as well as from NLRP3^−/−^ and Caspase1/11^−/−^ mice with *T. gondii*, and treated infected cells with eATP (3 mM), which activates the P2X7 receptor (26). Light microscopy-based quantification of the percentage of infected cells and the infection index (which combines the proportion of infected cells and the number of parasites per cell) showed that treatment with eATP decreased the parasite load in wild-type macrophages, while the parasite load remained high in cells from NLRP3^−/−^ and Caspase1/11^−/−^ mice (Figure 1). These data suggest that, in macrophages, the control of *T. gondii* infection mediated by eATP depends on the assembly of the NLRP3 inflammasome.

We demonstrated previously that P2X7 receptor activation reduces *T. gondii* load in macrophages, while also generating ROS (26). Given that, in macrophages, P2X7 receptor activation is associated with activation of the NLRP3 inflammasome (15), we sought to determine if ROS and caspase-1—the hallmark of the canonical NLRP3 inflammasome—are required for the reduction of *T. gondii* burden in BMDMs, *via* P2X7 receptor signaling.

**Figure 2:**
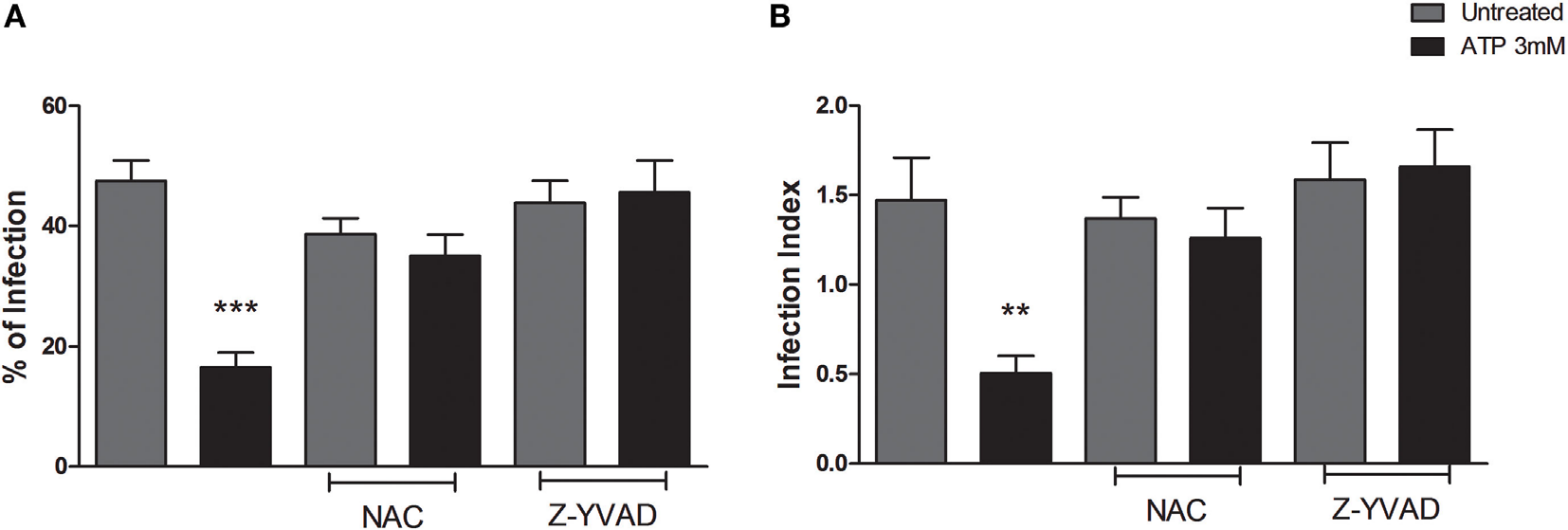
The decrease in *T. gondii* infection upon extracellular ATP (eATP) treatment depends on reactive oxygen species (ROS) generation and caspase-1 activation. Wild-type (C57BL/6) bone marrow-derived macrophages were infected with *T. gondii* tachyzoites for 2 h and then left untreated, or subjected to one of the following treatments: 10 mM *N*-acetyl cysteine (NAC) or 2 µM Z-YVAD for 30 min followed by 3 mM eATP, for 30 min. After treatments, cells were incubated for a further 18 h at 37°C, and stained with Panoptic, for light microscopy quantification of the percentage of infection **(A)** and the infection index **(B)**. Treatment with NAD or Z-YVAD before exposure to eATP prevented the eATP-dependent reduction in *T. gondii* infection. Data represent mean ± SEM of three independent experiments performed in triplicates. **p ≤ 0.01 and ***p ≤ 0.001 vs. untreated (by one-way ANOVA followed by Tukey’s test).

To address this issue, we infected BMDMs from C57BL/6 (WT) mice with *T. gondii* tachyzoites followed by pretreatment with NAC or Z-YVAD (which inhibit ROS and caspase-1 activities, respectively) and then treated infected cells with eATP. We confirmed our previous observations that eATP reduced the percentage of infected cells (Figure 2A) and the infection index (Figure 2B), in wild-type macrophage cultures. This effect was abolished by treatment with either NAC or Z-YVAD (Figure 2). Thus, the reduction in *T. gondii* infection load *via* eATP and the P2X7 receptor depends on activation of the canonical NLRP3 inflammasome, and also on ROS production and caspase-1.

### A Canonical, but Not a Non-Canonical, Inflammasome Mediates *T. gondii* Elimination *via* P2X7 Receptor Signaling

**Figure 3:**
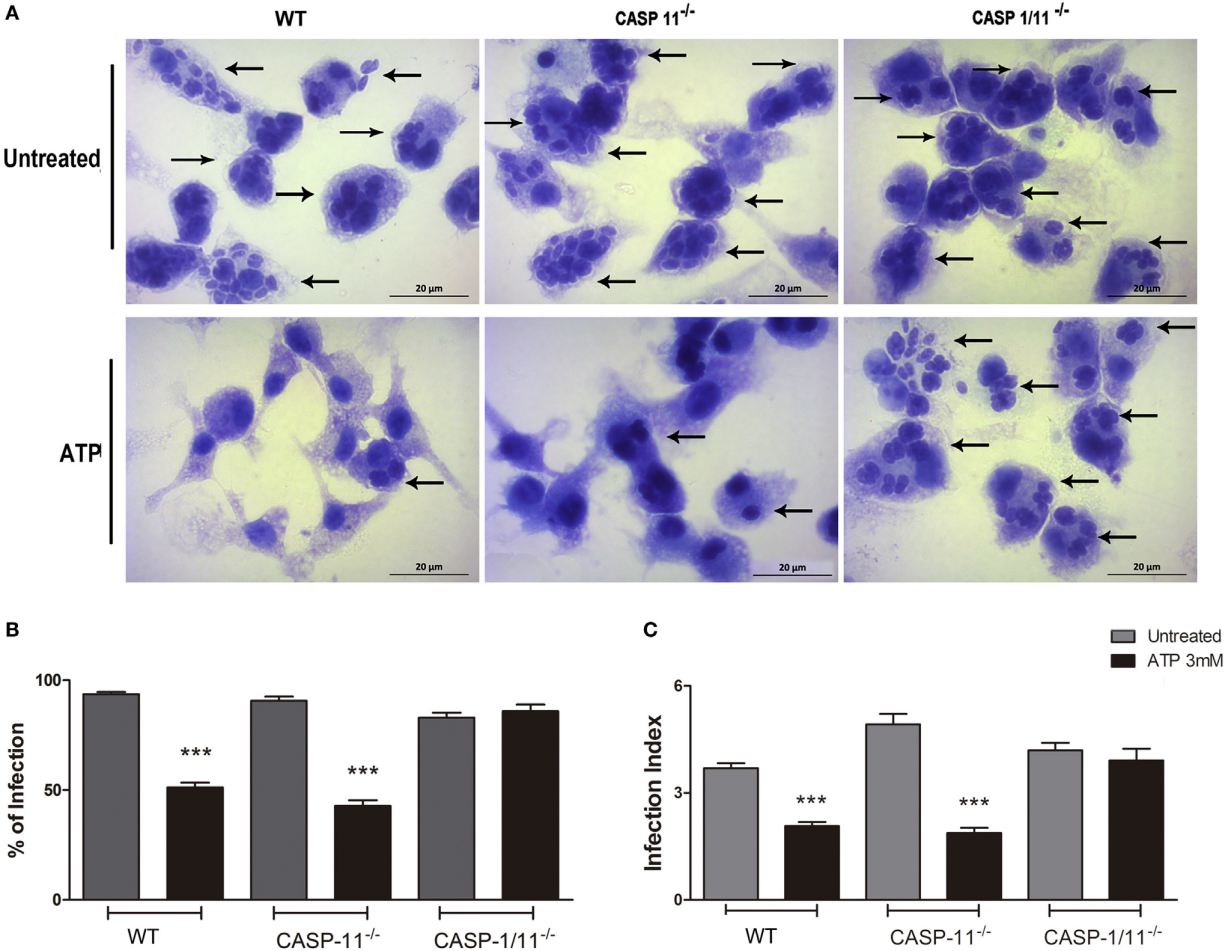
P2X7 receptor activation triggers *T. gondii* elimination *via* a canonical (but not *via* a non-canonical) inflammasome. Bone marrow-derived macrophages (BMDMs) from C57BL/6 (wild-type), and from Caspase-11^−/−^ and Caspase1/11^−/−^knockout mice (lacking non-canonical and canonical inflammasome effectors, respectively) were infected with *T. gondii* tachyzoites for 2 h and then kept untreated, or were treated 3 mM extracellular ATP for 30 min. Then, cells were incubated at 37°C for a total of 18 h, and stained with Panoptic, for light microscopy observation [**(A)**; representative images of three independent experiments] and for the quantification of the % of infection **(B)** and the infection index **(C)**. We observed reduced parasite load in wild-type and Caspase-11^−/−^ BMDMs, but not in Caspase-1/11^−/−^ cells. Black arrows indicate parasitophorous vacuoles containing *T. gondii* tachyzoites. Micrographs are representative of three independent experiments. Data represent mean ± SEM of three independent experiments performed in triplicates. ***p ≤ 0.001 vs. untreated (by two-tailed *t*-test).

Given that P2X7 receptor activation reduces *T. gondii* infection load in a caspase1/11-dependent manner (Figures 1 and 2), we investigated whether canonical and/or non-canonical inflammasome were involved in parasite elimination. To differentiate between canonical and non-canonical inflammasome contributions, we quantified the parasite load after eATP treatment in BMDMs from C57BL/6 (wild-type), Caspase11^−/−^, and Caspase1/11^−/−^ mice. Treatment with eATP reduced the parasite load in wild-type and Caspase-11^−/−^ macrophages, while no reduction in parasite load was observed after nucleotide treatment in Caspase1/11^−/−^cells (Figure 3). Thus caspase-1, but not caspase-11, is required for eATP-mediated *T. gondii* infection control. Combined with the data shown in Figures 1 and 2, these results confirm that the activation of the canonical NLRP3 inflammasome is crucial for the control of *T. gondii* infection in macrophages *via* P2X7 receptor activation by eATP. These data also show that caspase-11 is dispensable for pathogen elimination *via* eATP, while caspase-1 is required for this effect, defining a role for the canonical inflammasome only (and not for the non-canonical inflammasome) in *T. gondii* infection control mediated by purinergic signaling.

### eATP Induces NLRP3 Inflammasome-Dependent IL-1β Secretion and ROS Generation in Infected Macrophages

IL-1β is an important pro-inflammatory cytokine in response to different pathogens (37), and its secretion is induced by inflammasome activation (14). Therefore, we investigated whether activation of the canonical inflammasome *via* eATP/P2X7 receptor resulted in the secretion of IL-1β by *T. gondii*-infected cells.

**Figure 4:**
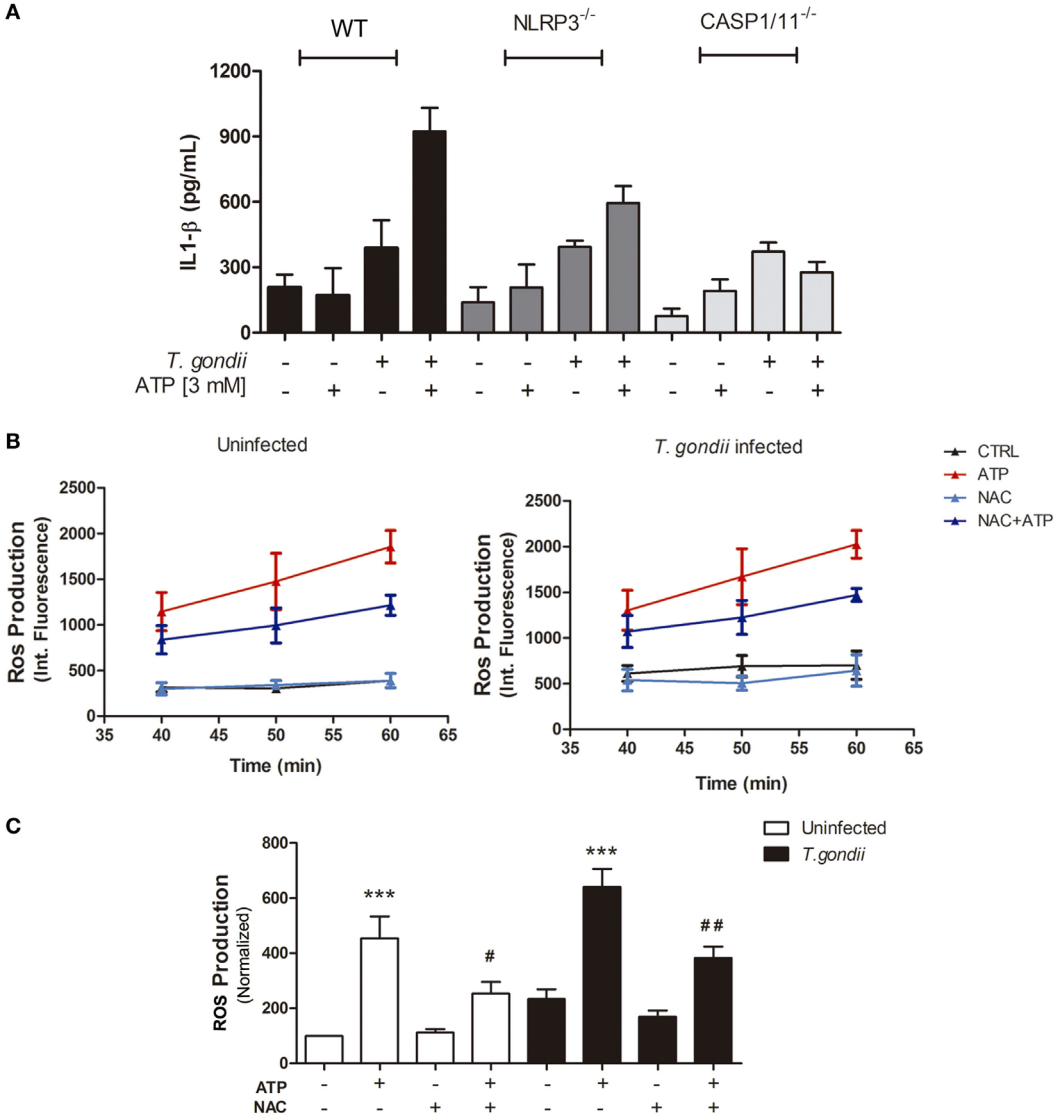
Extracellular ATP (eATP) is important for IL-1β secretion *via* canonical NLRP3 inflammasome induction and reactive oxygen species (ROS) production, in *T. gondii-*infected macrophages. Bone marrow-derived macrophages from C57BL/6 (WT), NLRP3^−/−^ or Caspase-1/11^−/−^ mice, either uninfected or infected with *T. gondii* tachyzoites, were kept untreated or were treated with ATP, and then culture supernatant samples were used to estimate IL-1β secretion **(A)**. Treatment with 3 mM eATP (for 30 min) was performed 2 h post-infection, and all cells were incubated for 24 h prior to IL-1β quantification. Data represent mean ± SEM from triplicates. For ROS detection, before eATP treatment, some cultures were incubated with 10 mM *N*-acetyl cysteine (NAC) and ROS production was detected. **(B,C)** ROS levels in cells treated for 30 min, with NAC and then with 3 mM eATP (for up to 60 min), 2 h post-infection. ROS levels were estimated by H_2_DCFDA fluorescence, at different time-points during treatment with eATP **(B)**, with normalized data from the 50-min time-point in **(C)**. Data represent mean ± SEM of three independent experiments performed in triplicates. ^#^*p* ≤ 0.05 and ^##^*p* ≤ 0.01 vs. corresponding samples treated with eATP only; ***p ≤ 0.001 vs. the corresponding untreated control (by one-way ANOVA followed by Tukey’s test).

*T. gondii* infection itself induced discrete (and similar) amounts of IL-1β release by WT (C57BL/6), NLRP3^−/−^ and Caspase1/11^−/−^ BMDMs (Figure 4A). When infected cells were treated with eATP, IL-1β secretion was potentiated in WT macrophages but did not increase in cells from NLRP3^−/−^ or Caspase1/11^−/−^ mice, showing that activation of the canonical NLRP3 inflammasome by eATP (*via* the P2X7 receptor) triggers IL-1β secretion in *T. gondii-*infected macrophages.

ROS production is one of the most potent microbicidal mechanisms against intracellular pathogens, and *T. gondii* efficiently block ROS production in order to survive and evade host immune mechanisms (38). In a previous study, we showed that treatment with eATP induces ROS production in murine macrophages (26). To test if *T. gondii* infection subverts eATP-induced ROS generation, we quantified ROS levels in *T. gondii-*infected peritoneal macrophages at different time-points (40, 50, and 60 min), after eATP. Exposure to eATP increased ROS levels, from 40 to 60 min of treatment (Figure 4B), even in *T. gondii*-infected cells (Figure 4B). The ROS level increase triggered by eATP after 40 min of treatment was reduced significantly by exposure to the ROS production inhibitor NAC (Figure 4C). Together, these results show that eATP induced the production of key molecules for the control of intracellular parasite infection—ROS and IL-1β—even in the presence of *T. gondii* and its evasion mechanisms.

### *T. gondii* Infection Control *via* eATP and IL-1β Depends on ROS Production and Pannexin-1

**Figure 5:**
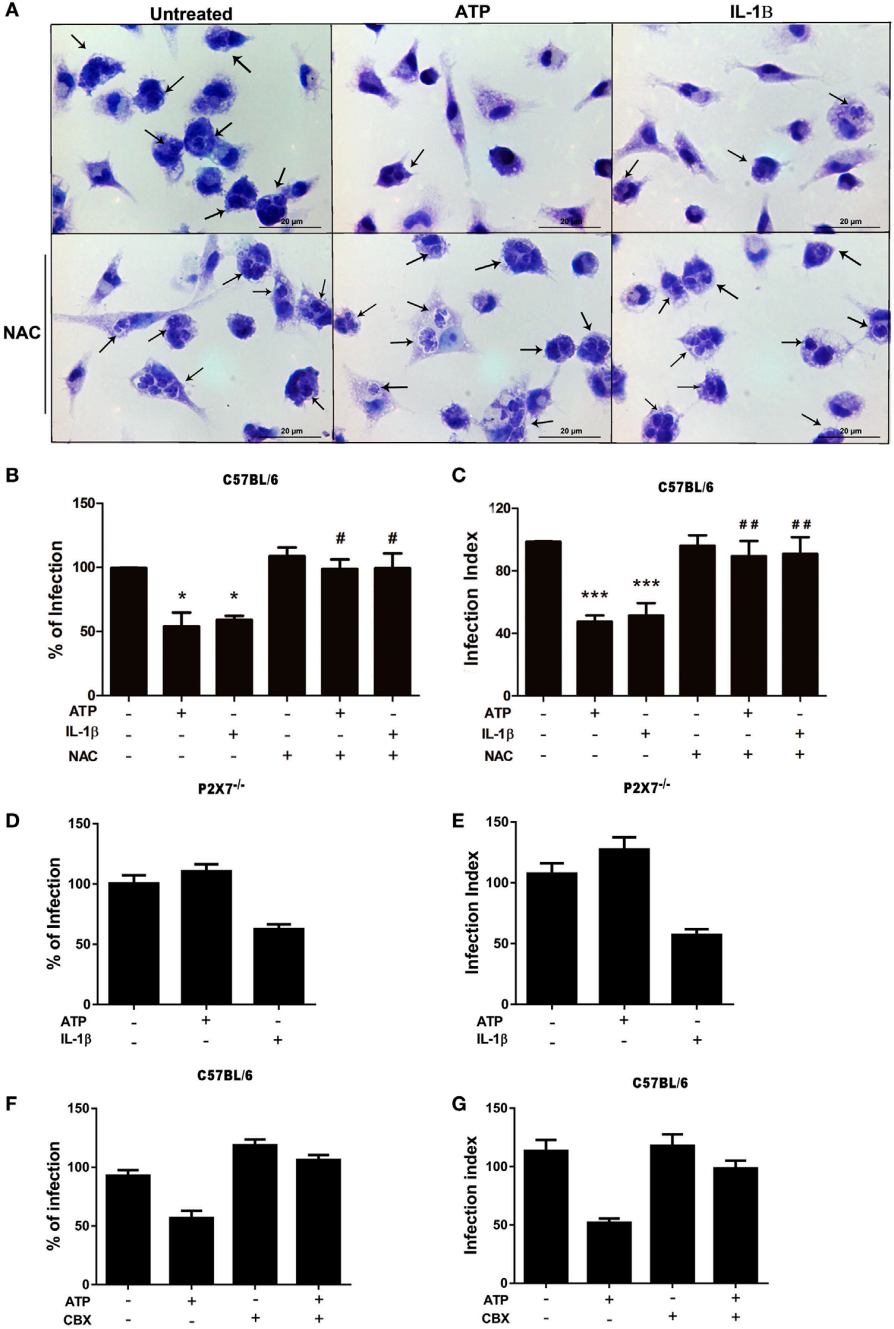
The control of *T. gondii* infection induced by extracellular ATP (eATP) and IL-1β requires the generation of reactive oxygen species and pannexin-1. Peritoneal macrophages from C57BL/6 (WT) and P2X7^−/−^ mice were infected with *T. gondii* tachyzoites for 2 h, and then left untreated or subjected to one or both of the following treatments as indicated: 10 mM *N*-acetyl cysteine (NAC) or 50 µM carbenoxolone for 40 min, followed by 3 mM eATP or 1 ng/mL IL-1β for 30 min. Then, cells were incubated at 37°C for a further 18 h, and stained with Panoptic for light microscopy analysis **(A)** representative images of three independent experiments, which was used to quantify the percentage of infection **(B,D,F)** and the infection index **(C,E,G)** in C57BL/6 **(B,C,F,G)** and P2X7^−/−^ **(D,E)** cells. In **(A)**, black arrows indicate parasitophorous vacuoles containing *T. gondii* tachyzoites in C57BL/6. Normalized data represent mean ± SEM of three independent experiments [in **(B–E)**] and 2 independent experiments [in **(F,G)**] performed in triplicates. **(F,G)** are representative experiments. Treatment with eATP or IL-1β reduced the parasite load in C57BL/6 cells, and this effect was abolished by NAC pretreatment **(B,C)**. Treatment with eATP had no effect in the parasite load in P2X7^−/−^ cells **(D,E)** or C57BL/6 cells pretreated with carbenoxolone **(F,G)**. ^#^*p* ≤ 0.05 and ^##^*p* ≤ 0.01 vs. sample treated with ATP only; *p ≤ 0.05 and ***p ≤ 0.001 vs. untreated (by one-way ANOVA followed by Tukey’s test).

Thus far, we showed that eATP treatment inhibits *T. gondii* infection in macrophages by a mechanism dependent on the canonical NLRP3 inflammasome and on ROS production, and that NLRP3 inflammasome activation in cells treated with eATP leads to the secretion of IL-1β. To examine in more detail the relationships between P2X7 receptor activation (by eATP), IL-1β production and ROS generation, during *T. gondii* infection control by macrophages, we treated infected peritoneal macrophages with eATP or recombinant IL-1β, alone or in combination with the antioxidant NAC. As expected, treatment with eATP or recombinant IL-1β reduced *T. gondii* infection in wild-type macrophages (Figure 5A). Interestingly, the antioxidant NAC completely inhibited the control of *T. gondii* infection by eATP and IL-1β in peritoneal macrophages (Figures 5B,C). These results show that ROS production induced downstream of P2X7 receptor activation or IL-1β secretion is essential for the control of *T. gondii* infection mediated by purinergic signaling.

Importantly, treatment with recombinant IL-1β reduced *T. gondii* infection load even in macrophages lacking the P2X7 receptor (from P2X7^−/−^mice) (Figures 5D,E). These data indicate that IL-1β is downstream of P2X7 receptor activation in the signaling network that results in *T. gondii* infection control by macrophages. In addition, these experiments confirmed that the effects of eATP treatment described here depends on the P2X7 receptor, since eATP treatment did not reduce the percentage of infected cells (Figure 5D) or the infection index (Figure 5E) in P2X7^−/−^ macrophages.

In order to reinforce the role of IL-1β promoting parasite killing, we assessed if the blockade of pannexin-1 channel, which is required for IL-1β secretion in macrophages (39) can inhibit eATP-induced killing of *T. gondii*. As shown in Figures 5F,G, carbenoxolone pretreatment abolished the eATP-induced control of the parasite load. These data confirm that eATP-induced IL-1β secretion from macrophages is crucial to *T. gondii* elimination.

### *T. gondii* Elimination *via* eATP and IL-1β Depends on Distinct ROS Generation Sources

**Figure 6:**
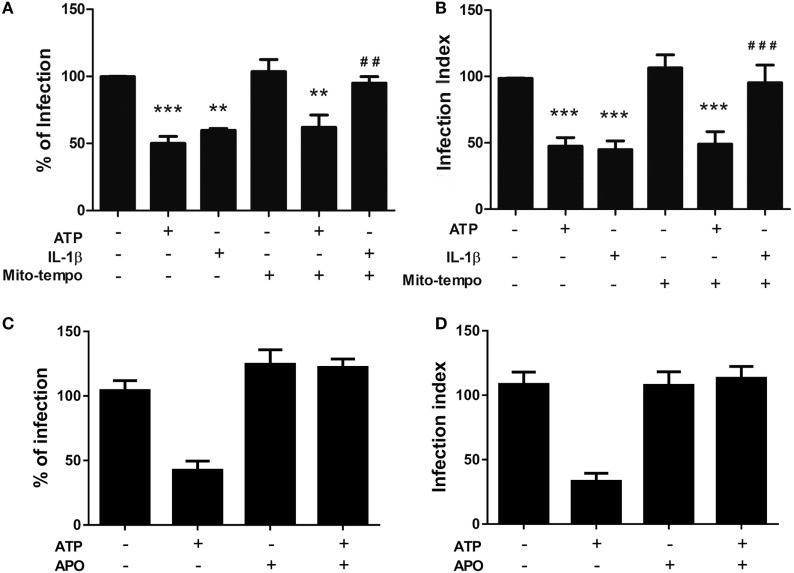
The control of *T. gondii* infection induced by extracellular ATP (eATP) and IL-1β depends on different sources of reactive oxygen species generation. Peritoneal macrophages from C57BL/6 mice were infected with *T. gondii* tachyzoites for 2 h and left untreated or subjected to one or more of the following treatments: 100 nM Mito-TEMPO or 1 µM apocynin for 40 min, followed by 3 mM eATP or 1 ng/mL IL-1β for 30 min. Then, cells were incubated at 37°C for a further 18 h, and the percentage of infected cells **(A,C)**, and number of parasites per host cell **(B,D)** were estimated by light microscopy examination, after staining with Panoptic. Normalized data represent mean ± SEM of three independent experiments performed in triplicates in **(A,B)** and two independent experiments in **(C,D)**. ^##^*p* ≤ 0.01 and ^###^*p* ≤ 0.001 vs. sample treated with ATP only; **p ≤ 0.01 and ***p ≤ 0.001 vs. untreated (by one-way ANOVA followed by Tukey’s test).

The precise role and origin of the ROS pool that acts as an anti-parasitic effector during P2X7 receptor activation remain poorly defined. Considering that our data support the notion that P2X7 receptor activation induces ROS generation *via* eATP treatment, we decided to examine which source of ROS pool is required for *T. gondii* infection control triggered by eATP, and whether this phenomenon is mediated by the canonical inflammasome effector IL-1β. To examine this question, we pretreated infected peritoneal macrophages with Mito-TEMPO, an inhibitor of mitochondrial ROS production, and then treated infected cells with eATP or recombinant IL-1β. Treatment with Mito-TEMPO effectively inhibited the control of *T. gondii* infection by IL-1β, while eATP treatment still reduced the parasite load even in the presence of Mito-TEMPO (Figures 6A,B). Then, we pretreated infected peritoneal macrophages with apocynin, an inhibitor of NADPH oxidase ROS production, and treated infected cells with eATP. Treatment with Apocynin completely blocked the eATP-induced reduction of parasite load (Figures 6C,D). Therefore, while both eATP and IL-1β reduce *T. gondii* infection load *via* ROS generation (Figure 5), the mitochondrial ROS pool is important only for the effector activity of IL-1β on *T. gondii* infection control. IL-1β-independent pathways of *T. gondii* elimination triggered by eATP are likely to depend on ROS production *via* NADPH oxidase.

## Discussion

**Figure 7:**
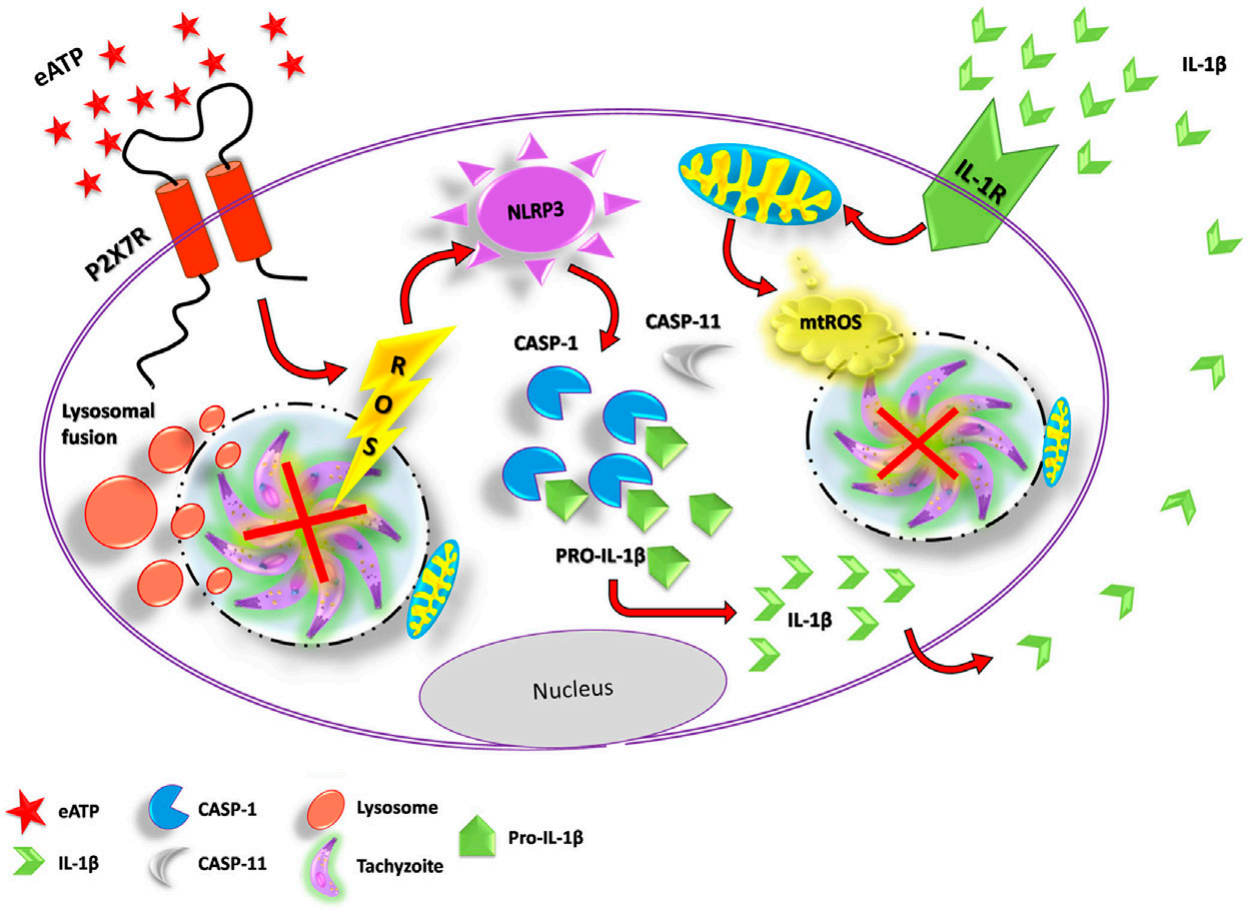
Proposed mechanism of *T. gondi* infection control mediated by extracellular ATP (eATP)/P2X7 receptor and IL-1β, in macrophages. Binding of eATP to the P2X7 receptor may lead to pathogen elimination *via* different mechanisms: the fusion of lysosomes to the parasitophorous vacuole (forming a phagolysosome), reactive oxygen species (ROS) generation and the activation of the canonical NLRP3 inflammasome. NLRP3 inflammasome activation by eATP potentiates IL-1β release through caspase-1 activity and, in turn, IL-1β in the extracellular compartment binds to its receptor, triggering *T. gondii* elimination *via* mitochondrial ROS generation.

Toxoplasmosis is a major parasitic disease transmitted by food, with a widespread distribution and a global human infection rate of ~30% (1). As toxoplasmosis is an inflammatory disease, the killing of *T. gondii* requires both innate and adaptive immune responses (40). Several lines of evidence—from gene polymorphism data to mouse knockout analysis—support the notion that P2X7 receptor activity contributes to control toxoplasmosis infection *in vivo*, by triggering antimicrobial activities in the intracellular environment (such as ROS production and lysosome fusion to the parasitophorous vacuole), and by stimulating pro-inflammatory events, such as the production of IL-12, IL-1β, and IFN-γ (27, 32). However, the downstream pathways that lead to parasite elimination *via* P2X7 receptor activation had not been dissected previously. Here, we described the cellular pathways that contribute to *T. gondii* killing induced by P2X7 receptor activation, showing that the control of *T. gondii* infection triggered by purinergic signaling requires the activity of a canonical NLRP3 inflammasome, and involves the inflammasome-dependent production of ROS and IL-1β (Figure 7).

IL-1β secretion, ROS production, and cell death are the most common cellular events linked to P2X7 receptor activation, and also seem to depend on inflammasome activation (20, 29, 35). ATP, a P2X7 receptor ligand, acts as a second signal to activate the NLRP3 inflammasome (41). Both NLRP1 and NLRP3 inflammasomes are involved in the immune responses against *T. gondii* infection, and mice lacking the inflammasome effectors caspase-1 and caspase-11 have 95% mortality after *T. gondii* infection (42). In agreement with these data, we show here that the control of *T. gondii* infection by P2X7 activation in macrophages depends on the NLRP3 inflammasome, and that the inflammasome effector caspase-1 is essential for *T. gondii* infection control by the P2X7 receptor, because lack of inflammasome components (NLRP3 and caspase-1) or inhibition of caspase-1 activity by Z-YVAD abolished the reduction in parasite load after treatment with ATP. In addition, the release of IL-1β in NLRP3 deficient mice can be explained by activation of NLRP1 inflammasome in macrophages of those mice (42). By examining separately the contributions of caspase-1 and caspase-11 to *T. gondii* infection control (using mouse knockout cells) we also show that the reduction in infection load caused by ATP treatment requires only caspase-1, and not caspase-11, supporting the hypothesis that a canonical NLRP3 is activated by the P2X7 receptor, to reduce *T. gondii* infection in macrophages.

Pyroptosis was shown to occur in response to P2X7 activation in macrophages (43), and this cell death mechanism can contribute to the clearance of intracellular pathogens (44). We did not detect significant cell death after 30 min or 18 h of eATP treatment (data not shown). In addition, our data suggest that, as have been shown for several pathogenic bacteria (45), the infection with *T gondii* may block pyroptosis. Thus, it is possible to conceive that different intracellular pathogens, not only bacteria, may subvert the immune response by operating in the inhibition of pyroptosis.

Even before it interacts with host cells, *T. gondii* secretes molecules that downregulate PRRs and, consequently, reduce the production of pro-inflammatory mediators (46). PRR activation acts as a first signal for inflammasome activation, inducing the transcription of genes coding for pro-inflammatory molecules. When PRR activation is followed by a second signal, it culminates in the cleavage and secretion of IL-1β (47). Our data show that upon eATP treatment and activation of the P2X7 receptor, *T. gondii* is not capable of downregulating the inflammasome-induced IL-1β secretion efficiently, since we observed that eATP treatment promotes IL-1β secretion in macrophages from WT mice. In this context, it is already described that P2X7 receptor activation leads to pannexin-1 pore formation and it is important for IL-1β release from macrophages (39). Our data show that inhibiting pannexin-1 channels, eATP-treated macrophages lack the ability to control *T. gondii* infection, which suggests that eATP-induced IL-1β secretion is crucial for the parasite killing. The results shown here reinforce the notion that IL-1β secretion potentiates the microbicidal effect of P2X7 activation.

Reactive oxygen species production is another key mechanism of intracellular pathogen elimination (48). *T. gondii* posses a specialized arsenal of antioxidant molecules and can successfully neutralize oxidative stress in the host (38). However, our result show that eATP treatment induced ROS production in murine macrophages even during *T. gondii* infection. Importantly, treatment with the inflammasome final product IL-1β reduced *T. gondii* infection load in macrophages, and ROS production was required for *T. gondii* infection control by eATP and IL-1β, because the pan ROS inhibitor NAC prevented the parasite load reduction caused by ATP and IL-1β. Our data show that, upon eATP treatment, ROS production is maintained during *T. gondii* infection, suggesting that P2X7 receptor activation overcomes sophisticated antioxidant mechanisms triggered by the pathogen. In phagocytes, NADPH oxidase activity and mitochondria are the two most common sources of ROS involved in immune response events (48), and the mitochondrial-derived ROS also activates the NLRP3 inflammasome (49). Here, we found that ROS derived from mitochondria participates in the control of *T. gondii* infection downstream of IL-1β signaling only, while IL-1β-independent signaling triggered by eATP is capable of controls *T. gondii* infection even upon inhibition of mitochondrial ROS production (by Mito-TEMPO). Therefore, we consider that NADPH oxidase-derived ROS are involved in the control of *T. gondii* infection in macrophages, upon P2X7 signaling activation.

## Conclusion

Our data demonstrate that eATP treatment reduces *T. gondii* infection load in macrophages through activation of the NLRP3 inflammasome, and that eATP promotes IL-1β secretion, and leads to ROS production, in infected cells. By using knockout mice for different inflammasome molecules, such as NLRP3 and caspase1 and 11, we also identified that a canonical (but not a non-canonical) inflammasome is involved in the eATP-induced IL-1β secretion and elimination of the intracellular parasites, in macrophages.

We conclude that P2X7 receptor activation inhibits *T. gondii* growth *via* ROS generated, most likely, from NADPH oxidase (rather than mitochondrial ROS), while also activating the canonical NLRP3 inflammasome, which leads to IL-1β secretion and infection control *via* mitochondrial ROS production.

## Ethics Statement

The procedures for the care and use of animals were according to the guidelines of the Brazilian College of Animal Experimentation (COBEA). All efforts were made to minimize animal suffering and to reduce the number of animals used in this study. This study was approved and followed all the guidelines established by the Ethics Committee on the Use of Animals (CEUA) of the Biophysic Institute Carlos Chagas Filho (IBCCF, UFRJ, no. 082/15).

## Author Contributions

ACAMS and CLCAS designed and performed the experiments, analyzed the results, and wrote the manuscript. TPR and GCR performed experiments and analyzed the results. MB, DZ, and RCV analyzed the results and revised the manuscript. RCS designed the experiments, analyzed the results, and revised the manuscript.

## Conflict of Interest Statement

The authors declare that the research was conducted in the absence of any commercial or financial relationships that could be construed as a potential conflict of interest. The reviewer DD-R declared a shared affiliation, with no collaboration, with several of the authors, AM-S, TR, and RV, to the handling editor.
